# Intermittent Fever, Part 1a

**Published:** 1891

**Authors:** P.P. Wells


### INTERMITTENT FEVER.

By this term we mean a disease, the nature of which is to appear in paroxysms which recur at intervals with intervening periods of freedom from the paroxysmal phenomena, these periods being of various duration in different cases. The paroxysms, when perfectly expressed, are made up of four elements—the phenomena of the circulation, chill, heat, and perspiration—which appear in succession, the three last in the order named, and have, in the complete form we are now contemplating, a certain symmetry of proportionate duration and intensity in relation of each to the other. This disease is the result of the action of a specific poison on the human organism. It attacks all classes and conditions of men who are exposed to its action with perfect impartiality. The duration of its effects is not subject to any self-imposed limitation, but continues till the life of the patient is destroyed or the action of the poison is conquered by appropriate means. But the manifestations of the presence and action of this poison are not always met in the perfect, symmetrical paroxysms. On the contrary, these are oftener than otherwise very irregular, either in the comparative duration or intensity of the different paroxysmal elements, or any one or two of these may fail of appearance in any given case, or these may change the order of their appearance in any possible order of succession, or any two of these elements may be mixed, appearing at the same time, or may be alternated in repeated succession for a longer or shorter time. Or, instead of the fever as described, there may be, as a result of this poison, a variety of affections, more or less painful and annoying, appearing as rheumatism, neuralgia, dysentery, etc. But in whatever form the effects of this poison may appear, it is a peculiarity of each that it is characterized by this one feature of periodicity.

The origin of this poison was formerly charged to the presence of stagnant water in the infected locality, especially as this is found in swamps and marshes. But it chanced there were many of these localities where there were no cases of ague. Then decomposing vegetable matter was added as another factor in its production, and these two combined were credited with the origin of the whole evil. But observation has fully established the fact that there are many localities where these are both present, and yet there are no malarial fevers. And more than this, some of the localities where the fever is met in its most malignant form have neither of these elements present as a generating cause. Such are the Walcheren district, in Holland, and the high, arid desert in Central Spain, and many others. So that, while it is known and admitted that these two are active agents in producing this poison in some localities, there are others where they are absent, and yet the fever is there met at its worst. It follows, then, that there are other factors producing this poison, of which at the present time but little is known beyond the facts which result from its action. And more than this, there are localities where, after the absence of agues from time immemorial, they suddenly appear, while all external circumstances, so far as these can be appreciated, remain the same as they have ever been. And then it has happened, where agues have so suddenly appeared, the generation of the poison has extended in a given direction, say from west to east, as of late years on the north shore of Long Island Sound, each year adding new territory to the dominion of the disease, till large districts of country are invaded which but lately were wholly exempt from the plague. Such facts plainly declare that, as to the etiology of ague, there is much still remaining unknown. It is not uncommon to refer the facts just stated to telluric influences of some sort, which only leaves the whole subject in the same darkness as before, in which, notwithstanding this attempted explanation, it is likely to remain till it can be further shown what is the kind and nature of this influence so charged. At present, certainly, we know nothing about this branch of causation, and we should know nothing of the existence of the poison so mysteriously produced but that its existence is revealed to us by its effects. The prevalence of the poison is more common in warm latitudes than in cold, and yet in these there are many localities where there is no ague. Where it is present, its production and the intensity of its action are favored by increase of heat, as well as in swampy districts, by drying the surface, which is usually wet, and by its being wet again after being dried. In such localities the fall of rain is often followed by increase of agues. It is notable that though in these cases water seems to play so important a part in the production of the cause of these fevers, yet they prevail in others where there is little or no water, and where this agent can be supposed to have had little to do in the production of their cause. A notable example of this is met in the high sandy plains of Spain. In this and the dry localities of Italy and Greece the fall of rain is followed by abundant malaria. This has been attributed to the ammonia present in the water of the rain-fall, which is, of course, merely hypothetical.

It is also worthy of remark that in many localities, notably in mountainous regions, in spite of frequent exposure to chills, and of extreme changes of temperature from hot days to cold nights, and the greatest exposure of persons, there are no intermittent fevers, while, on the contrary, localities where the greatest evenness of temperature prevails, the sickliest months are those which show the least variation.

The production of the poison is modified by the quantity of water present in the infected locality. Where the water is deep it is less, and more where it is shallow.

The facts stated above and many others show conclusively that while water, decomposing organic matter, and high temperature are active factors in the genesis of ague poison in many instances, in others, where these are mostly or wholly absent, the poison is present and active in a notable degree. From this it may be accepted with confidence that there are other factors which originate the poison in question, either conjointly with these, or, in some localities, apparently independent of them. As to just what these last factors are, we may speculate as much as we please, and at the end remain in the same ignorance as at the beginning. We know they exist and act by reason of their effects, which are only too apparent. Beyond these we know little or nothing of them. How far these unknown factors may be responsible for the great variety exhibited in the symptomatic combinations of the resulting fevers, by their impress on, and modification of the results of the action of the better known causes of ague poison, can only at present be matters of conjecture. That the poison is not, in each instance of its presence, an identity, represented fully in every other specimen wherever met is clearly indicated by the varied character of the phenomena of resulting fevers, and more clearly still by the varied specific relations of these to their respective curatives. How far a knowledge of the nature and characteristic action of the unknown factor or factors may hereafter explain the known facts that agues contracted in different localities, even in those of near neighborhood are not cured by the same remedy, in the epidemics of the same year, for example, those contracted at the opposite ends of a given lake, as has been observed of one in Lombardy ; or the fact that agues of the same locality are not cured by the same remedy in the epidemics of succeeding years, is a question for the future to solve. But experience has already taught, by a multitude of examples, that these facts have their place in the history of intermittent fevers, as this has grown from the observations of those who have seen most of them and have seen them best. This difference of susceptibility to the action of curatives plainly declares a difference of identity in the fevers of different localities and epidemics. Though all have the defining characteristics which place them properly in the family of intermittents, and by which they are related to this family, they differ in the specific characteristics by which they are related to their curatives. It does not matter that defining symptoms in successive cases so like to each other as to make distinction difficult or impossible, and that therefore it has been concluded that the same remedy must be a cure for all, for it is not in this class of symptoms that the law of cure finds the elements with which it has to do. It is inevitable that a practice so founded will meet with more of disappointment than success. For though the intelligence of the practitioner may fail to detect the distinctions which relate his case to its specific curative, the keener perceptions of the living susceptibilities of the organism will not, neither will they respond curatively to remedies not so related, let popular or professional opinion, prejudice, or expectation, be what they may. Then it follows that the idea that Cinchona or any of its constituent elements, or any other drug or nostrum, is or can be a specific cure for this class of fevers, is without foundation in truth or in sound medical philosophy. The variety in the causation of the poison is followed by a corresponding variety in its results—the fever—and variety in this last calls for a corresponding variety in the means of cure.

The poison finds its way into the organism of those who come in contact with it through the absorbing surfaces which it meets, passes through these to those organs and tissues for which it has special affinities, and upon these and through these it works its special mission of destruction.

Its first impression seems to be made, as might be expected, on the nervous centres. The manifestation of this is various in different cases. In uncomplicated intermittent the attack is often initiated by fever without preceding symptoms intimating its approach. In other cases there is found an introductory stage of simple debility, with slight febrile phenomena, without symptoms as yet of any localization in any particular organ. With these there may be headache, loss of appetite, coated tongue, pressure in the epigastrium, nausea, and vomiting. In other cases with the early febrile symptoms there may be greater debility, with continued gastric pains, confusion and heat of the head, vertigo, accelerated pulse, and dark-colored urine. In other cases there are violent pains in the limbs. This state may continue six, eight, or even as long as ten or twelve days before the appearance of the fully developed characteristic paroxysm. During this stage the spleen may become somewhat swollen and sensitive to pressure. The face pale or of an earthy color, and the *bruit de diable* may be heard in the large veins of the neck. This may be followed by a succession of slight chills which increase in violence till they come to the fully developed paroxysm, or strong chill may be followed by the heat and sweating which complete the series of stages of the complete paroxysm. This is succeeded by a remission of symptoms, or by those which may be characteristic of the fever, and when recognized are of the utmost importance as guides to the simillimum the law demands at the hand of the prescriber.

After this period of remission, which may vary greatly in its duration in different cases, the paroxysm repeats itself, and the process is fully set up in the organism which results in a series of these which return at regular or irregular intervals, with little tendency in the disease to find its limitation in length of time, or in any number of repetitions of the paroxysms. There is little tendency to self-exhaustion of its power in the cause of these repeated phenomena.

The object of this paper is to help, if we may, those who need help to a right treatment in accordance with the homœopathic law and philosophy. In endeavoring to carry out this object we have given a brief consideration to its etiology and symptomatology. We have seen that the cause, produced in different circumstances, generates fevers composed of elements very various in their intensity of action, as well as in the combination of these in their paroxysmal manifestation, these differences in the elements of the paroxysms and in their varied combination as to intensity, duration, or absence of one or more of them in cases, as well as the time, condition, and circumstances of their manifestation, together with the concomitants of each element of the paroxysm, these, with the development of morbid phenomena in the intervals of the paroxysms are the facts with which we have to do in our search for the curing agent of the case. It is with these and not with the diagnostic name, or with defining elements of the case which have given to it its name that we are to be chiefly concerned. Loyal dealing with these, as required to constitute any treatmeut of a case *homœopathic*, will speedily and certainly relieve the practitioner of foolish notions of the insufficiency of our law and the means it employs for the cure of this often perplexing and troublesome malady. It cannot be denied that these are very common exhibitions of weakness in those who call themselves of our school, but who have understood and embraced our law but partially, and have but partially acquainted themselves with its scope, and the powers of the agents it employs for cure, and are therefore easily led to mistake this personal defect in themselves for insufficency of the law and its means when they fail to cure intermittents.

We hear this complaint of "inefficiency" often from this class of practitioners, and are not in the least surprised when we hear it. Indeed, it is difficult to see how it could have been otherwise with them than that they should fail. Being to so great extent ignorant or rejecters of our law and its corollaries, and equally defective in knowledge of the means it employs, as well as of what is comprised in this familiar expression of a fundamental factor in *all* homœopathic prescribing—"*the totality of the symptoms*"—being ignorant of the extent of the meaning of this phrase, they are of necessity wholly incapable of compassing this fundamental element in all homœopathic prescribing, and of course they fail to cure. They do not know *how* to go to work to find this, and so, instead of with this in hand, or trying to gather it, they are content with the few generic symptoms—chill, heat, and sweating—and with these they proceed to give same one or *more* of the hundreds of drugs which have these more or less in their pathogenesis, and, of course, fail. And, therefore, Homœopathy has "failed to cure ague." Homœopathy has had no more to do with the whole proceeding than has the 119th Psalm. Then, having failed in this proceeding, they have one perennial resort—*i. e.*, to some one of the drugs which has power to suppress the paroxysmal phenomena of the disease, and after their kind of investigation, it is not surprising that they "generally find this to be *Quinine*." The paroxysm suppressed, and they cry—Behold a cure of which one "may brag a little."* Of all cures which are not cures, these so bragged of are the most pernicious.

We have said the practice described is not homœopathic. If it

---

* See description of this subject in *Hahnemannian Monthly*, April, 1882.

be asked why is it not, we reply, because it is wanting in the first element in every homœopathic prescription, without which the homœopathicity is wanting, here and always, viz.: "*The totality of the symptoms.*"

We will now try to see in what this "*totality*" consists in the disease we are now concerned with. We shall find it in four general divisions :

1st.—The circulation.

2d.—Chill.

3d.—Heat.

4th.—Sweating.

To find this "totality" each of these is to be examined as to its especial disturbances and their concomitants, and these each and all, as to time of manifestation, causes of aggravation, and relief, as well as to all other circumstances and conditions which modify these phenomena. We shall give some of these, with a view to showing the extent of this examination, leaving the names of the medicines connected with them to be sought by the searcher in the materia medica.

In the first place, then, of the *circulation*, we have distention of the veins generally, of those of the head, of the face, of the throat, on the hands, on the feet. Burning in the veins, inflammation of the veins, cold sensations in the veins, throbbing, varicose veins, marbled appearance of capillaries.

*Congestion*—in general ; to the head ; eyes ; ears ; nose, nose bleeding ; face ; chest ; abdomen ; upper extremities ; lower extermities.

Plethora.

Anemia.

Sensation of obstructed circulation ; agitation of the circulation.

*Heart*—palpitation of, in general, with anxiety ; intermittent heart-beating. Heart beat—shaking ; fluttering ; felt (sensible) ; audible ; visible ; trembling.

*Pulse*—intermittent ; accelerated ; thread-like ; tense ; large ; frequent ; hard ; audible (to patient) ; jerking ; small ; slow ; quick ; quick in the morning, in the daytime or evening slow ; quick in the afternoon, slow in the morning; quick in the evening; quick at night, slow in the daytime; weak; strong; imperceptible; unequal; irregular; gone; unchanged; full; soft; trembling; jerking.

*Time* of aggravation of the disturbances of the circulation, as morning, forenoon, afternoon, evening, night.

*Conditions of aggravation* of the disturbances of the circulation. Prostrated mental condition; after anger; in the paroxysms; from bodily exertion; from rising up; from motion; from stooping; before sleep; on waking; from vomiting; before eating; while eating; after eating; while walking; while walking in the open air; after walking in the open air; from mental emotions; from coughing; from lying in bed; from lying on the back; lying on the left side; lying on the right side; before the catamenia; during the catamenia; from music; after lying down; in repose; in sleep; in sleep in the afternoon; from sleeplessness; while sitting; while stooping; from speaking; from standing; in a warm room; after stool; from tobacco smoke or from smoking; going up-stairs; from drinking; from drinking beer, brandy, coffee, tea, wine; from turning in bed; from warm weather.

Such are the facts in relation to the circulation which are to be inquired into in every case of intermittent fever. That such of them as are present in the case to be treated may be brought to light and given its true place and importance in the selection of the one specific, which is the objective of every true homœopathic prescription, the finding of which is the end of the duty of every true homœopathic physician in every clinical effort.

The second general division of the "totality of the symptoms" is the *chill.*

This second division is to be dealt with in the same manner as to detail and analysis as in the first, and the first fact to be inquired into is, is the chill predominant? Then is it *external* only? Does the skin present goose-flesh with this? Is it *one-sided?* Is it *left* or *right* side? Chill which *runs downwards* or *upwards; internal chill;* chill with *shaking;* chill running over the whole surface; chill with trembling; *slight* chilliness;

*coldness in general; partial coldness;* in the upper part of the body; in the lower part of the body; in front; in back of the head; proceeding from the head; from the back; in the head; on the ears; on the nose; on the face; proceeding from the face; of the lips; in the epigastrium; proceeding from the epigastrium; in the hypochondrium; in the abdomen; going from the abdomen.

*Partial coldness*—of the neck; of the chest; going from the chest; of the shoulder-blades; going from the shoulder-blades; of the back; going from the back; of the loins; going from the loins; upper arms; going from upper arms; of the fore arms; of the hands; going from the hands; of the fingers; of the lower extremities; of the thighs; of the knees; legs below the knees; of the feet; going from the feet.

*Chilliness in general*—partial; one-sided; on the back of the body; on the left side; on the right side; on the suffering part; on the head; on the ears; on the nose; on the face; on the cheeks; on the lips; on the chin.

*Coldness*—in the mouth; of the tongue; in the epigastrium; in the abdomen; of the genitals; of the glans penis; of the testicles; in the chest; on the back; on the loins; proceeding from the loins; of the upper extremities; of the hands; of the fingers; of the ends of fingers; of the lower extremities; of the thighs.

*Partial coldness*—of the knees; of the legs below the knees; of the feet; of the toes.

*Sensation of coldness in general*—local sensation of coldness on the head; in the head; in the eyes; in the eyelids; in the ears; on the face; on the face, one-sided; on the lips; on the chin; in the teeth; in the mouth; in the throat; of the tongue; in the stomach; in the hypochondria; in the abdomen; in the genitals; in the trachea; on the throat and neck; in the chest; on the chest; on the shoulder-blades; on the back; on the loins; on the upper extremities; on the hands; on the fingers; on the ends of fingers; on the lower extremities; on the right lower extremity; on the thighs; on the knees; on the legs below the knees; on the feet; on the toes.

*Shivering in general*—with goose flesh ; one-sided ; ascending ; descending ; here and there, wandering ; internal ; running over the body.

*Partial shivering*—on the head ; going from the head ; on back of head ; on the face ; going from the face ; on the chin ; in the epigastrium ; in the hypochondria ; in the abdomen ; on the scrotum ; over throat and neck ; over the chest ; over the shoulder-blades ; over the back ; going from the back ; over the loins ; on the upper extremities ; going from the arms ; on the lower extremities ; on the knees ; on the legs.

*Time of exacerbation*—morning ; noon ; forenoon ; afternoon ; evening ; night ; before midnight ; about midnight ; after midnight ; daytime ; recurring at the same hour ; about three o'clock in the afternoon ; evening ; between four and eight o'clock ; every fourteen days ; recurring at the same time of the year.

*Conditions of exacerbation*—after anger ; after each paroxysm ; from taking hold of cold objects ; from undressing or uncovering ; from rising up ; from rising from bed ; in bed ; from being touched ; touching cold objects ; from moving ; after moving ; after vomiting ; after being heated ; after waking ; before eating ; while eating ; after eating warm food ; after paroxysms of epilepsy ; in the open air ; while yawning ; while walking in the open air ; after walking in the open air ; before urinating ; while urinating ; after urinating ; from coughing ; in the cold air ; before catamenia ; during catamenia ; after catamenia ; from warm stove ; in sleep ; after sleep ; during the pains ; after the pains ; during coryza ; after fright ; during vertigo ; in the side on which one lies ; while sitting ; while speaking of unpleasant subjects ; in a warm room ; before stool ; during defecation ; after defecation ; after drinking ; from turning in bed ; after taking cold ; after taking cold by being wet through ; from alternation with mental symptoms ; from alternation with pains ; from damp, cold weather ; from stormy weather ; from pains in the teeth ; from wind current.

*Circumstances which relieve*—after arising from bed ; while in bed ; from motion ; after eating ; in the open air ; from walking in the open air; while lying down; after sleep; when sitting; in the sunshine; in a warm room; from drinking; from external warmth.

*Concomitant symptoms—Disposition*—anxiety of mind; irritability; indifference; serenity; melancholy; discouraged; dejected; depressed spirits; sadness: super-sensibility; insensibility; restlessness of mind; angry irritability; despairing; disposition to weeping; rage.

*Intellect*—dullness; insensibility, loss of consciousness; delirium; giddiness; confusion (*eingenommenheit*); ecstasy; weakness of memory; delusions; vertigo; staggering; intellect excited; intellect weak; madness (insanity); empty-minded.

*Headache*—with rush of blood to head; burning in the head; throbbing; jerking; shooting; bursting pain; contracting pain. External head—swelling; hair standing on end (sensation of); sensitiveness of scalp; heat; sweat.

*Eyes*—burning; pressure; inflammation; sparkling; pupil dilated; pupil contracted; pains in the eyes; staring; shooting; tears; burning of lids; twitching of lids; swelling of lids; dryness of lids. Vision—movings before eyes; flames; flickering; photophobia; clouded vision; dimness of vision; darkening of vision; disappearing of the power of vision; trembling before the eyes.

*Ears*—pains; pressure; heat; heat of external ear; redness; shooting; hearing sensitive; ringing; roaring; deafness.

*Nose*—bleeding; pressure; heat; itching; redness; dryness.

*Face*—swelling; pale; bluish; purple; earth-colored; yellow; pale red; one-sided redness; also with coldness, and also alternating with coldness; heat; coldness; convulsions; perspiration; pains; tension; distortion.

*Lips*—eruptions; swelling; dryness.

*Teeth*—chattering; grinding; painful.

*Mouth*—burning in the mouth; dryness; offensive smell from mouth; pain in throat; increased saliva; coated tongue; dry tongue; aversion to food; nausea from food; hunger; thirst for beer; for stimulating drinks; thirst; before the chill; between the chill and the heat; thirstlessness; bitter taste; flat taste; foul taste; metallic taste; salt taste; sour taste; sweetish taste; loss of taste.

*Eructations* — tendency to vomiting; vomiting; bitter; bloody; food; sour; slimy; black; watery; nausea; heart-burning; water-brash.

Pain in stomach; pain in liver; pain in spleen; pain in kidneys; pain in hypogastrium; swelling of the abdomen; coldness in abdomen.

*Diarrhœa*—painful; painless.

*Constipation*—from intestinal inactivity; from hard feces; urgency to stool; tenesmus.

*Urgency to urinate*—unsuccessful urgency; too frequent urination; painful; seldom urination; involuntary urination; retention of urine.

*Sneezing*—coryza; fluent; dry.

Respiratory affections; suffocating attacks; deep inspiration; breath hot; cold; slow; loud; without mucus rattle; rattling; rapid; sighing; irregular.

*Cough*—with expectoration; without expectoration.

*Larynx*—hoarseness.

*Neck*—stiffness; pains.

*Chest*—stitch; warm sensation; palpitation.

*Shoulder-blades*—shootings.

*Back*—pains in; in the loins; lameness.

*Upper extremities*—pains in; hands as if dead; swelling of the veins; blue; heat; fingers as if dead; heat; blue nails; carphologia.

*Lower extremities*—pains; hips; thighs; knees; legs; toes; feet as if dead; swelled; heat; coldness; bodily exhaustion; swelling of the veins; nervous excitability; the limbs as if asleep; loss of sensation; spasms, clonic or tonic; crawling; lameness; weakness; subsultus; faintness; drawings in the muscles; in the joints; in the bones; bending; stretching the limbs; heaviness of the limbs; shootings in the joints; stiffness in joints; restlessness; the body as if bruised; internal trembling; jerkings; drawing the limbs together.

2

*Skin*—blueness ; burning : burning in ulcers ; yellow skin ; itching ; shooting in skin ; sensations of contraction in skin ; yawning ; sleepiness ; loss of sleep ; in sleep starts with fright ; sliding down in bed ; murmuring ; snoring ; talking ; groaning and whimpering.

*Heat*—dry heat, as if hot water was poured over him.

*Partial heat*—one-sided ; left side ; right side ; fore part of the body ; back part ; upper part ; lower part ; parts covered ; heat on the head ; proceeding from the head ; in the head ; on the eyes ; eyebrows ; eyelids ; in the corner of the eyes ; on the ears ; in the ears ; proceeding from the ears ; on the external ears ; in the face ; proceeding from the face ; on the forehead ; on the cheeks ; on the cheeks, one-sided ; on the uncovered cheek ; on the pale cheek ; on the nose ; in the nose ; running from the nose ; on the lips ; the upper lip ; the under lip ; on the under jaw ; on the chin ; in the mouth ; streaming from the mouth ; on the palate ; heat in the throat ; on the tongue ; in the teeth ; on the gums.

*In the stomach*—proceeding from the stomach ; on the epigastrium ; heat in the region of the liver ; heat in the region of the spleen ; heat in the region of the kidneys ; heat in the abdomen ; in the upper part of abdomen ; in the lower part of abdomen ; heat of abdomen (external) ; heat proceeding from the region of the umbilicus ; in the inguinal region ; on the perineum ; on the anus ; in the rectum ; in the urinary bladder ; in the urethra ; in the male genitals ; on the prepuce ; on the glans ; on the penis ; on the scrotum ; in the testicles ; in the spermatic cord ; on the female genitals ; in the vulva.

*In the larynx and trachea*—on the throat ; on the nape of the neck.

*In the chest*—in the region of the heart ; on the chest (external) ; in the mammary glands ; in the axillæ ; on the shoulder-blades ; in the back ; in the loins ; on the coccyx.

*Heat of the upper extremities*—in the shoulders ; on the shoulder joints ; upper arm ; elbow ; forearm ; wrist ; hands ; on one hand ; proceeding from the hands ; on the back of the hand ; the palm ; the fingers ; ends of fingers.

*Lower extremities*—heat on the hips; in the hip joint; on the buttock; the thighs; knees; legs; shinbone; on the calf; ankle; the feet; proceeding from the feet; the heel; on the back of the feet; the sole; the toes; ends of the toes.

### Aggravation of Heat.

*Time*—morning; forenoon; afternoon; evening; night; before midnight; after midnight; four o'clock P. M.; from six to eight o'clock P. M.; recurring at the same hour; in short, repeated paroxysms; slow, rising to its maximum, and slowly declining; slow in reaching its maximum, and suddenly disappearing; suddenly appearing and disappearing.

*Conditions*—after anger; during work; after rising from bed; after coition; in bed; during motion; after motion; from drinking beer; from stooping; from uncovering; from vomiting; waking from sleep; before eating; while eating; after eating; from eating meat; riding in a carriage; after breakfast; while walking in the open air; after walking in the open air; from noise; from hand working; from coughing; drinking coffee; in the climacteric period; from intellectual efforts; from reading: lying in bed; before the catamenia; after catamenia; during the catamenia; suppressed catamenia; in sleep; after sleep; after noontide siesta; during pains generally; during coryza; while sitting; in the sunshine; from speaking; from standing; in a room; before stool; at stool; after stool; from smoking tobacco; drinking water; from wine; after working; while teething (children); from being covered.

*Conditions and circumstances which alleviate*—after supper; bodily exercise; from leaving the bed; in bed; from moderate motion; from drinking beer; from stooping; from uncovering; after vomiting; after awaking; while eating; after eating; while riding in a carriage; after breakfast; while walking in the open air; from drinking coffee; from loosening one's clothes; from intellectual labor; during sleep; while sitting; while standing; in a room; after stool; from smoking tobacco; from working; from washing the face; from drinking water.

*Concomitant symptoms; disposition*—anxiety; excited disposition ; active ; sensitive to noise ; indifference ; impetuous ; serenity ; complainings and lamentations ; weariness of life ; melancholy ; misanthropy ; ill humor ; discouragement ; depression of spirits ; disposed to whistling ; loquacious ; silent ; fearful ; screaming ; suicidal disposition ; sighing and groaning ; singing and trilling ; spitting ; fear of death ; sadness ; excessive sensibility ; restlessness ; angry ; despairing ; changeable disposition ; disposition to weeping ; whimpering and whining ; rage.

*Intellect*—dullness ; loss of consciousness ; delirium ; anxious delirium ; loquacious delirium ; serene delirium ; with muttering ; silent delirium ; violent delirium ; giddy ; confused ; dullness ; excited imagination ; illusions ; vertigo ; staggering ; drunken dizziness ; intellect excited ; insanity ; wildness.

*Pains in the head*—in the occiput ; with congestion ; heaviness.

*Scalp*—perspiration on forehead ; cold sweat ; tension.

*Pains in the eyes*—swelling around eyes ; blue borders around eyes ; burning ; protruding ; pupils dilated ; contracted ; redness ; strabismus ; dryness ; diminished power of vision ; darkness before eyes ; sparks ; flimmering ; green color before the eyes ; photophobia.

*Pains in the ears*—coldness of ears.

*Hearing*—rushing ; roaring , deafness.

*Pains in nose*—itching ; coldness.

*Face*—swelling ; pale ; brownish-red ; earth-colored ; yellow ; one side red ; red on the uncovered side ; circumscribed red ; cold face ; coldness of cheeks ; of the forehead ; sweating ; cold sweating ; pains in the face.

*Lips*—eruptions ; swelling ; dryness ; swelling of sub-maxillary glands ; pains in teeth ; chattering of the teeth ; bleeding of the gums ; swelling of gums ; pain in gums.

*Mouth*—burning ; offensive smell ; yellow around the mouth ; dryness.

*Throat*—pains ; burning ; inflammation of the uvula ; dryness ; increased saliva ; coated tongue ; dry tongue ; speech difficult.

*Food*—aversion to food : aversion to drink ; disgust for food ; disgust for drink; canine appetite; desire for beer; desire for cold drinks; desire for acids; for cold water; for wine.

*Thirst*—between the chill and the heat; thirst, with aversion to drinking; thirst for large quantity at a time; for little at a time; thirstlessness; thirstlessness, with desire to drink.

*Taste*—bitter; putrid; salt; bad.

*Eructations*—disposition to vomiting.

*Vomiting*—bitter; foul; sour; slimy.

*Nausea*—water-brash.

*Stomach*—pains; trembling sensation; burning; pressure; cramps.

*Liver*—pains; pains in *spleen*; swellings.

*Abdomen*—pains; swelling; cold sensation; squeezing; throbbing; tension; labor-like.

*Flatulence.*

*Diarrhœa.*

*Constipation*—urgency to stool; urgency to stool without result.

*Urine*—brown; stinking; turbid; too small quantity; too large; too often; too seldom; painful urination; urgency to urinate; fruitless urgency.

*Sneezing*—fluent coryza; dry coryza; dryness of the nose.

*Respiration*—anxious; oppressed; hot; cold; short; rattling; deep. *Cough*—with expectoration; without.

*Larynx*—Pains; dryness; hoarseness.

*External* pains in throat; throat sensitive; swelling of glands; stiffness.

*Chest*—internal pains; sensation of rising in; congestion; cramps; shootings; constriction; swelling of mammary glands. Loss of milk; palpitation of heart, with anxiety.

*Shoulder-blades*—pains.

*Back*—pains; in loins; in coccyx.

*Upper extremities*—pains; in joints; hands dead; swelling of veins; blue; cold; sweat; trembling; jerking; retraction of thumbs; dead fingers; cold.

*Lower extremities*—pains; heaviness; restless; pains in hip, thigh; numbness; pains in knees; coldness; pain in legs; feet dead, swollen, cold; coldness of one foot.

Sweating of feet; relaxation; swelling of veins; burning in; throbbing in; nervous excitement; covering unendurable; limbs asleep; disposition to uncover; aversion to uncovering; covering insupportable; carphologia; loss of feeling.

*Limbs in general*—pains; clonic spasms; tonic spasms; crawling in limbs; paralysis; disposition to lie down, weakness; jerking of muscles; fainting; drawings; in the joints; in the bones; turning and stretching; apoplexy; heaviness of the limbs; shootings in muscles; in the joints; in the bones; bodily restlessness; bruised sensation in limbs; tremblings; jerkings.

Swelling of glands.

Eruptions on the skin; sweating; pale; burning; yellow; itching; crawling and pricking; parchment like; redness; shooting; dryness; bones; pains.

*Sleep*—stretching; yawning; sleepiness; sleep between chill and heat; stupefying; sleeplessness.

*In sleep*—waking with fright; sliding down in bed; murmuring; snoring; groaning and whimpering; dreams.

*Sweating*—which breaks out easily; perspiration wanting; suppressed; sensation of perspiration breaking out; anxious perspiration; perspiration which causes smarting; smelling like musk; bitter smelling; bloody; smelling like blood; empyreumatic smells; burning sweat; musty smelling, debilitating; not debilitating; putrid smelling; smelling like spoiled eggs; oily; which shrivels the fingers; which spots the linen; which attracts flies; stains the linen yellow; stains the skin and eyes yellow; without smell; smells spicy; hot; smelling like elder; smelling like honey; smelling like cheese; cold; smelling like camphor; sticky; with sensation of crawling; cadaverous smelling; shining; mouldy smelling; smells like horse urine; like rhubarb; red perspiration; which stains red; sour smelling; pungent smelling; smelling like sulphur; like sulphureted hydrogen; which stiffens the linen; sweet smelling; sweetish acid smelling; stinking; smells like urine; like wheat bread; stains cloth white; excoriating; smelling like onions.

*Partial perspiration*—only on the head; only on the head at night; on the upper part of trunk; on the lower; one sided; left; right; anterior; posterior; only on itching parts; on painful parts; on single small spots; on joints; on parts on which one is lying; on covered parts; on uncovered parts; on one side of the head; on the occiput; cold sweat on the head; sticky; on the ears; on the nose; on the face; proceeding from the face; one side of face; on the forehead; cold on face; on the forehead; on upper lip; on the epigastrium; on abdomen; proceeding from the navel; on the groin; about the anus; on mons veneris; on perineum; on male generative organs; honey-like smelling on these organs; offensive on ditto; on the scrotum; one-sided on scrotum; on female sexual organs; on the throat; on neck; on chest; cold on chest; offensive on chest; in armpits; offensive in the axillæ; on the back; on the whole arm; on the forearm; on the hands; cold on hands; sticky on hands; on the palms; on the fingers; on whole legs; on thighs; on knees; on legs below the knees; on the feet; proceeding from the feet; cold on feet; offensive on feet; suppressed on feet; excoriating on feet; on the soles; on or between the toes.

*Time of perspiration*—morning and forenoon; afternoon; evening; night; before midnight; after midnight; perspiration more in daytime; recurring periodically; in frequent; short attacks; every other day; recurring at the same hour.

*Circumstances*—anger; before the attack; during the attack; after the attack; during bodily exertion; on leaving the bed; on closing the eyes; with suppressed secretions; after coition; in bed; on motion; after moving; before falling asleep; while sleeping; on waking; after waking; while eating; after eating; from warm food; during an epileptic attack; after an epileptic attack; after an attack of fever; in the open air; while walking; while walking in the open air; during hard labor; before urinating; while urinating; after urinating; while coughing; after itching of the skin; in the cold air; in the climacteric period; during mental effort; while lying down; among strangers; in the beginning of the catamenia; during the catamenia; after lying down; in repose; before sleep; in the beginning of sleep; during sleep; with the pains; during coryza; after fright; with vertigo; while sitting; from speaking; in a room; before stool; after stool; from smoking tobacco; while dreaming; while drinking; from warm drinks; on waking; in the wind; during toothache; from being covered.

*Circumstances which relieve*—exertion of the body; exertion of mind; after rising from bed; from motion; after motion; while going to sleep; from uncovering; after waking; while eating; after eating; while walking in the open air; while lying in bed; in repose; during sleep; after sleep; from speaking; in a room; after stool; after drinking water; after drinking wine; after working.

*Concomitant symptoms*—*Disposition*—anxiety; excitability; sensitive; indifference; impetuous; serenity; complaints and lamentations; suicidal disposition; melancholy; misanthropic; sadness; discouraged; depressed; loquacious; silent; tearful; cries; sighing and groaning; singing and humming; fear of death; sadness; super-sensitive; impatient; restless; morose; despairing; changeable disposition; disposition to weeping; whimpering; rage.

*Intellect*—dullness; unconsciousness; delirium; giddiness; confusion of intellect [*eingenommenheit*;] excited imagination; vertigo; intellect excited.

*Pains in the head*—internal; external.

Pains in the eyes; pupils dilated; pupils contracted; vision diminished; sparks like fire; flimmering; photophobia.

Noises in the ears; pains in the ears.

Pains in the nose; itching in the nose; cold nose.

Swelling of the face; face pale-bluish red; shining, as if greasy; yellow; red, hot; cold; cold cheeks; cold forehead; pains in the face. *Lips*—eruptions; swelling; dryness; swelling of submaxillary glands.

Pains in teeth; bleeding of the gums; swelling of gums.

*Mouth*—burning; offensive smell; dryness.

*Throat*—pains; burning; inflammation; inflammation of uvula; dryness; increase of saliva; tongue coated; tongue dry.

*Loss of appetite*—disgust for food; hunger. *Thirst*—between the heat and the perspiration; after perspiration; no thirst; bitter taste; putrid taste; salt, foul eructations; disposition to nausea; vomiting; bitter vomiting; of food; sour vomiting; of mucus; nausea; water gathering in mouth.

Pains in stomach; in the liver; in the spleen; in the abdomen; flatulence; diarrhœa; constipation; urging to stool; fruitless efforts to stool.

*Urine*—pale; brown; offensive; turbid; deficient; excessive; too frequent; too seldom; painful; suppressed; urging to urinate; fruitless urgency.

Sneezing—fluent coryza; dry coryza; dryness of nose.

*Respiration*—anxious; oppressed; hot; cold; short; rattling; deep. *Cough*—with expectoration; without. *Larynx*—pains; dryness; hoarseness.

*Throat*—pains; external; sensitive; swelling of glands; stiffness of neck; pains in neck.

*Chest*—pains; sensation of rising up in chest; congestion; heart-beating. *Mammary glands*—swelling; milk increased; milk diminished.

Pain in *shoulder-blades, back*, loins, *coccyx*.

*Superior extremities*—pains; pains in the joints; hands dead; swelling of veins; blue; heat; cold; trembling; jerking; thumb retracted; fingers dead; fingers hot; fingers cold; fingers wrinkled; nails blue.

*Inferior extremities*—pains; heaviness; restless; pains in the hips; pains in thigh; coldness; pain in knees; coldness of knees; pains in legs; dead feet; swelling of feet; feet hot; feet cold; lassitude; swelling of the veins; burning in veins; throbbing of veins; nervous excitement; limbs asleep; disposition to uncover the limbs; uncovering them insupportable; carphologia; loss of sensation; pains in limbs in general; cramps; crawling in the limbs; lameness; disposition to lie down; weakness; jerking of muscles; faintness; drawing in muscles; drawing in joints; drawing in bones; stretching and twisting of limbs; apoplexy; heaviness of the limbs; shooting in the muscles; in the joints; in the bones; bodily restlessness; limbs as if bruised; trembling; jerking.

Swelling of glands.

*Skin*—eruptions; smarting; burning; itching; crawling and prickling.

*Bones*—pains.

Yawning; sleepiness; sleep; sleep after the perspiration; coma; sleeplessness; in sleep, fright; in sleep, expiration, blowing; sliding down in bed; muttering to one's self; snoring; groaning and whimpering; dreams.

The above catalogue of symptoms is given to illustrate the *extent* of the meaning of the phrase so often, so easily, and too often so thoughtlessly used by those who suppose they have compassed this extent after only the most superficial and brief examination of cases. They proceed to select and give medicine for their cure, and what they regard as the homœopathic principle of similars, when the elements of the case by which it is related to its curative have not been brought to light. They have not been seen. And no man can be certain he has seen them till in his examination he has gone over *all* aberrations of function and sensation possible in the case. We refer to the expression, "TOTALITY *of the symptoms*." The catalogue here given shows the extent of investigation necessary before there can be any certainty that the required "totality" is compassed in a case of intermittent fever, and therefore any certainty that those symptoms are discovered which relate the case to its curative. We have translated this catalogue of rubrics from Bœnninghausen's *Homœopathischen Therapie du Fieber*, omitting the names of the medicines given under each, the object being rather to show the extent of the field of inquiry than to present a repertory of symptoms of medicines and disease involved in the *homœopathic* treatment of this fever. It is but little less significant of the extent of the inquiry in the necessary investigation which must precede the homœopathic treatment of any and every other form of disease, and of each and every case of it before the prescriber can be certain he has found his required simillimum. It may be there will be no symptoms found in many of these rubrics, in a case to be prescribed for, but this can only be known after the search. Our object is to show how extended this search must be if duty be not neglected, and so far as we may be able to show how this is to be prosecuted in order to demonstrate the folly and the falsehood of the oft-repeated "*Homœopathy will not cure ague!*" It may, indeed, be true that those who have never tried the true method of homœopathic prescribing cannot cure ague with medicines in form and doses such as are used by the ordinary practice of our school. But it does not follow, because *they* cannot cure, that therefore *Homœopathy* cannot. Our endeavor is to show that it can, and how we are to proceed in order to demonstrate this fact.

We have said the elements which constitute a paroxysm of this fever are seen in all possible variety of combination in practice. The following are some of the forms which are not infrequently met with in treating agues.

*Paroxysms beginning with Chill.*

Chill, then heat.
Chill, then sensation of heat.
Chill, then heat of individual parts.
Chill, then heat of face.
Chill, then heat of head.
Chill, then heat with thirst.
Chill, then heat without thirst.
Chill, then heat without thirst and without sweat.
Chill with thirst, then heat.
Chill with thirst, then heat without thirst, then sweat.
Chill with thirst, then heat with thirst, then sweat.
Chill without thirst, then heat with thirst.
Chill without thirst, then heat with, then sweat without thirst, then heat with thirst.
Chill without thirst, then heat without thirst.
Chill, then heat and both with thirst.
Chill, then heat, then chill with thirst.
Chill, then heat, then sweat.
Chill, alternating with heat, with thirst, then sweat.
Chill, then heat, then sweat, with thirst.
Chill, then heat, then sweat without thirst.

Chill, then heat, then sour sweat.
Chill, then heat, with sweat.
Chill, then heat without sweat.
Chill, then heat with sweat on face.
Chill, then heat with internal chill, then heat and sweat.
Chill with, then heat without thirst.
Chill, then heat, then sweat.
Chill with heat at the same time.
Chill with heat and external heat.
Chill with heat and flashes of heat.
Chill with internal heat.
Chill with internal heat and sensation of heat.
Chill with internal heat and thirst.
Chill and heat, both internal.
Chill of some parts, with heat of others.
Chill with heat, without thirst.
Chill with heat, with thirst.
Chill with heat, then sweat.
Chill, then sweat, without previous heat.
Chill, then cold sweat.
Chill, then sweat, without heat and thirst.
Chill, then sweat, then heat.
Chill, then sweat, then thirst.
Chill, then thirst, then sweat.
Chill, alternating with heat.
Chill, alternating with heat, then heat.
Chill, alternating with heat, then sweat.
Chill, alternating with sweat.

*Beginning with Heat.*

Heat, then chill.
Heat, then chill, then heat, then sweat.
Heat, then coldness.
Heat of the face, then chill.
Heat, then shuddering.
Heat of the face, then shuddering.
Heat of the head, then coldness, then heat.

Heat, then sweat.
Heat, then cold sweat.
Heat, then sweat, then thirst.
Heat, then sweat, then heat.
Heat with chill and shuddering.
Heat with chill and shuddering and thirst.
Heat with chill, and shuddering without thirst.
Heat with chill, and shuddering, then sweat.
Heat with internal chill.
Heat with external coldness.
Heat with coldness of single parts.
Heat with sweat.
Heat with sweat and thirst.
Heat with sweat without thirst.
Heat with sweat and thirst, then chilliness.
Heat and thirst, alternating with chill.
Heat, with thirst, then sweat.
Heat in the head, alternating with chilliness of the legs.
Heat, alternating with shuddering.
Heat, with sweat, then chill.
Heat, with sweat and external coldness, then chill, then heat and external coldness.
Heat, alternating with sweat.

*Beginning with Shuddering.*

Shuddering, then chill.
Shuddering, then chill, without thirst.
Shuddering, then chill, then heat, without sweat.
Shuddering, then heat.
Shuddering, then heat, with chill.
Shuddering, then heat, with thirst.
Shuddering, then sweat.
Shuddering, with heat.
Shuddering, with heat of face, without thirst.
Shuddering, with sweat.
Shuddering, alternating with heat.

*Beginning with Sweat.*

Sweat, then chill.
Sweat, then chill, then sweat.
Sweat, then heat.
Sweat, alternating with dry skin.

If, after the view we have attempted to present of the origin, character, and constitution of this paroxysmal fever, we are called to its treatment, where shall we begin, and how proceed, if we are to deal with it as required by the law of similars? What are the facts in the case to which we are to bring the simillimum which cures? We answer, first, we are carefully to gather all the departures from healthy action of function and sensation which have *preceded* the paroxysm. Especially note whatever of aberrations there may be in the functions of the organs of circulation. Write them down. Note with which of the constituent elements of the paroxysm of the fever this is inaugurated. Then add to this record the facts of the paroxysm, beginning with the *time* of the appearance of its initial phenomena, then the exact locality of this first appearing. Then, was the first element, chill, heat, or sweat? Then, if chill, how did it appear, as external, internal, or both? In what *direction* did it progress from this initial point? Was the chill general or partial? If partial, what parts are affected? Was it simple coldness, or was it accompanied by shaking, shivering, or shuddering? Was the chill preceded, accompanied, or followed by thirst? Was the chill simple, or mixed with the other elements of the paroxysm? If mixed, with which, and in what order of combination—*i. e.*, are these elements co-existent, or in alternation? If the chill be with thirst, is it for cold or warm drinks? How, if at all, is the chill affected by drinking? What functions are most disturbed or perverted before, during, or immediately after the chill? In answering this, especial attention is to be given to the cerebral, gastric, and respiratory functions as well as to the circulation.

Having the record of the chill and its concomitants as above suggested, the facts accompanying the heat are to be examined in the same manner as to its beginning (time and locality), direction of its progress, and all accompanying phenomena, giving especial attention to *thirst* in its relation to this element. Then, is the heat simple, or mixed with chill or sweating? If so, in what order? Is the mixture alternating or concomitant? Is the heat, as compared with the chill, predominant in duration or intensity in the paroxysm? What are the functions of life most affected and perverted during this stage of the paroxysm, and how?

Then if the paroxysm begins with sweating, this is to be examined in like manner, in detail as to all its concomitants, and modes, and with especial attention to its relation to thirst; the parts most affected, the character of the perspiration; is it hot, warm, or cold? If it be recognized by the sense of smell, what is the character of the odor—*i. e.*, is it sour, sweet, bitter, moldy, or what is the character of the secretion in this respect, if it be none of the above? Then, when *each* of these elements of the paroxysms has been thus examined, which has been found to preponderate in intensity and duration?

All this is to be done before the prescriber asks himself the question—*What is the remedy for the case?* All this ground is to be gone over carefully in every case before any man, no matter how skillful, can answer this question as required by the law of similars. If, after this, the attempt to give a practical answer to this question results, as in case of one not long since engaged in a public discussion of this fever, "that this, (the homœopathic remedy) is, *in most cases*, *Quinine*," the prescriber may, without hesitation, pronounce on himself sentence of incompetency to deal with the problem before him. His patients and the public may very safely join in the confirmation of this sentence.

It is difficult to conceive of a greater absurdity than this, that a man recognizing the fact and authority of the law of *similars* as the law ordained for all healing, should, before a problem so complex in its origin, so variable and varying in its paroxysmal elements as we have found this fever to be, after such an examination of a case as is indispensable to his acquaintance with the facts which this law requires of him, that he shall find among *all* drugs that *one* which is most like the example before him, declare that he has found in *one* drug alone the required likeness of the almost infinite variety of facts and combinations of facts presented to us in this fever, or at least, that he "finds this in most cases!" Utter blindness and ignorance of all that constitutes a practice in accordance with our law in the treatment of this fever only can account for an utterance so absurd. Ignorance, it must be, both of the nature of diseases and of the drug action by which these are cured. This is quite apparent if we remember that however many facts in any given case, as we have attempted to suggest them, may be found in the pathogenesis of any one drug, there will remain a large and much larger number which are not. And in finding the similimum for the next dozen cases, we may be compelled to go beyond this drug, or the object of our search will not be found. Then this absurd statement further convicts its author of ignorance of the practical difference between suppression of the paroxysms of a disease and its cure. And yet, Heaven save the mark! its author was a "*teacher in one of our colleges!*" If this is the kind of stuff they taught it is no wonder the young men they graduate leave the school fully impressed with the falsehood, "*Ague and fever cannot be cured homœopathically!*"

It may facilitate finding our specific for our case if we have grouped together the elements of the paroxysms of this fever as they have been developed in the provings of different drugs. The study of these groupings is chiefly useful as aids to an intelligent differentiation of drugs which in this process have disclosed similar actions on the functions of different organs. It will be found, if the study be intelligent and thorough, that however similar these may be in some of the disturbances so caused, and however numerous these may be, showing clear and even intimate relationship of these drugs, there are other and equally important actions in which they differ. This, in materia medica, is a fact of utmost importance, as it should always be remembered these *differences* are the facts which individualize the drug, separating it from all other drugs, even those to which it has most resemblances, and declare to the prescriber the true, *specific* character of each.

It is then self-evident, in order to specific prescribing, which homœopathic prescribing always is, a precedent knowledge of this specific character of the agent to be employed in the cure is a *sine que non.* And this can only be attained by a knowledge, not of the *similarities* in drug action on the organism, but of the *differences.* To facilitate this a number of these groupings are here given from that master-work of that great master of homœopathic science and art, Bœnninghausen, in his last and best work, *Versuch einer Homœopathischen Therapie der Wechsel und anderer Fieber*, Leipsig, 1864.

In giving these groupings we shall select those most frequently called for, as we have found, in our rather extensive practical experience with this fever. We begin with

**Aconitum.**

1. Pulse for the most part very full, hard, and accelerated. Seldom small and thready, or imperceptible.—*Cold sensation in the veins.* [See Arsenicum.]

2. Chill at the beginning of the attack, most severe evenings after lying down; often with hot cheeks and contracted pupils. Chill from uncovering or from touch; with the chill often internal, heat with anxiety and red cheeks; shivering, extending from the feet to the chest.

3. Dry, burning heat, for the most part proceeding from the head and face, with great thirst for cold drinks; with the heat tossing about; continued heat, with disposition to be uncovered; uncommon excitement; restlessness; anxiety, and agonizing burning heat, with cold shiverings running over one at the same time.

4. Long-continued sweat over the whole body, of a somewhat acid odor; sweating most on parts covered.

Aconite may sometimes be found serviceable in relieving oppression of the heart and respiration when this is great during the paroxysm. It is the one exception to the rule which requires remedies for this fever to be given in the intermission.

Where this oppression is great, and the other symptoms not contraindicting it, a few pellets of the appropriate potence may be dissolved in half a tumbler of water, and a teaspoonful given every fifteen, twenty, or thirty minutes, according to the severity of the symptoms (the greater the oppression the shorter the intervals between the doses), till relief is obtained. This, if the remedy be in place, will not embarrass the action of the specific remedy for the case, though this may be another than Aconite. This remedy will be the more appropriate if the oppression be accompanied by the characteristic loud complaining and the equally characteristic fear of near death.

**Agaricus.**

1. *Pulse* in the morning somewhat accelerated, later in the day always slower; very irregular and sometimes intermitting.

2. Chill and chilliness predominant, especially in the cold, open air and while airing the bed; cold shuddering over the body from above downward.

3. Heat, slight, and almost only on the upper part of the body.

4. Fatty sweat, but not offensive, through the night in sleep; sweat from slight motion.

**Alumina.**

1. Pulse full and somewhat accelerated.

2. Chill predominant and for the most part toward evening, even in bed and by a warm stove, as well as after eating a warm soup, often with heat of the face; chill in the day and heat at night.

3. In the evening, following the chill, there is heat, which seems to spread from the face, but sometimes only attacking the right side of the body.

4. Sweat at night, especially in the bed; mornings, with anxiety; most on the face, and often only on the right side of the face; entire inability to perspire.

**Ammonium-carb.**

1. Pulse hard, tense, and rapid.

2. Chill in the evening, often alternating with heat, till midnight; chill in the open air.

3. Heat, most in the evening, with cold feet.

4. Sweat mornings, most on the joints; constant sweat, day or night.

**Ammonium-mur.**

1. Pulse constantly accelerated, day and night.

2. Chill, with external coldness, evenings and when uncovered in the night; chill every half-hour, alternating with heat; coldness runs up the back.

3. Heat, with red, swollen face, especially in a warm room and after bodily exertion; frequent flashes of heat, each ending in sweat, which is most on the face, palms of the hands, and soles of the feet.

4. Day and night sweat often preceding heat; copious night sweat over the whole body, most after midnight and mornings in bed.

**Anacardium.**

1. Pulse accelerated, with throbbing in veins.

2. Chill and coldness, with trembling, especially in the open air, disappearing in the sunshine; shuddering chill over the back, as if cold water were poured over it, with heat of face; internal chill, even in a warm room.

3. External heat with internal chill; heat of upper part of body, with cold feet; internal cold shuddering and hot breath, daily from four o'clock P. M. till evening, which disappears at supper time.

4. Evening, sweat on head, abdomen, and back; even when sitting still; night sweat on abdomen and back; sticky sweat on palms of hands, especially on the left; cool sweat, with internal heat.

**Angustura.**

1. Pulse accelerated, jerking, irregular, and sometimes intermitting.

2. Chill in the bed, morning and forenoon, after previous thirst; every afternoon (three o'clock) strong internal chill; repeated shuddering chills on the covered parts; forenoon (nine o'clock) shuddering chill on the back.

3. Evening, heat, most on face, after entering a room and after supper. After midnight (three o'clock) heat, which prevents sleep, with subsequent shuddering.

4. Sweat only in the morning and only on the forehead.

**Antimonium-crud.**

1. Pulse very irregular, now accelerated and now slow, alternating every few beats.

2. Chill predominates in the daytime, even in a warm room, toward noon severe shaking chill, with thirst (for beer); sensation of coldness in the nose while inspiring through it.

3. Heat predominates at night, but with cold feet till midnight; great heat from the slightest movement, especially in the sunshine.

4. Morning, when waking, sweat which shrivels the ends of the fingers, sweat which returns at the same hour, usually every other morning.

**Antimonium-tart.**

1. Pulse full, hard, and accelerated, sometimes trembling; strong pulsation of the veins; with the decline of the fever the pulse is often slow and imperceptible; on the slightest movement the pulse is uncommonly accelerated.

2. Chill, with external coldness predominant at all times of the day, with coma, mostly with trembling and shaking, and often as if water were poured over one; in the daytime, chill alternating with heat.

3. Great heat of short duration after a long chill, increased by every movement; long-continued heat after a short chill, with coma, and sweat on the forehead.

4. Copious sweat over the whole body, also in the night; sweat is often cold and sticky; the painful parts sweat constantly and most copiously.

**Apis-mellifica.**

1. Pulse full and accelerated, seldom small and thready, sometimes trembling and imperceptible.

2. Chill severest toward evening; afternoon (three or four o'clock), chilly shuddering, increased by warmth; chilliness from least movement, especially toward evening, with heat of face and hands.

3. Dry heat toward evening. The sensation of heat is greatest on the chest and epigastrium.

4. Sweat, alternating with dryness of the skin.

**Arnica-montana.**

1. Pulse very variable, mostly full, hard, and accelerated; pulse sometimes very weak and slow; in the evening, strong pulsation through the whole body.

2. Internal coldness with external warmth; chill, with great thirst, which often precedes it, for the most part in the evening, as if cold water were poured over one; chill and coldness of the lower parts of the body, with heat of the upper, especially of the head; universal chill, with heat and redness of cheeks; chill after every sleeping; chill from the least movement of the covering; chill, alternating with heat; cold sensation of the side on which one is lying;

3. Dry heat, which is either general or only running over the face and back.

4. Burning in single parts of the body, which feel cold externally, and heat or coldness, now here now there. Heat in the evening, with pains in the limbs.

5. Sweat for the most part smells sour, or is offensive; sometimes it is cold.

**Arsenicum-album.**

1. Pulse weak and small, but greatly accelerated, often imperceptible and entirely wanting, or intermittent. Pulse quick in the morning and slow in the evening. Burning or cold sensation in the veins.

2. Chill (and heat) indistinct, and either concomitant or alternating; chill in the forenoon, which nothing relieves; internal chill with external heat; chill and shuddering after every drinking; external coldness, with cold, sticky sweat; during the chill (and the heat), appear many concomitant symptoms in severity, which before were only of slight importance.

3. Internal burning, dry heat. Evenings and nights dry heat, with frequent thirst, but he drinks very little at a time; nocturnal heat, as if hot water were poured over one.

4. Sweat at the end of the fever, with disappearance of all symptoms, even the concomitants, and sweat in the first sleep or through the whole night; cold, sticky, or sour, or bad smelling sweat; unquenchable thirst during the sweat.

**Aurum-fol.**

1. Pulse small, but accelerated; much blood ebullition in the whole body, and strong congestion of head and chest.

2. Chill predominant; chill and coldness of hands and feet, also in bed, and continuing the whole night. Evening in bed general cold shuddering; coldness of the whole body, with nausea.

3. Heat, mostly in the face, alternating chill.

4. Sweat in the morning, most on genital organs.

**Baryta-carb.**

1. Pulse accelerated, but weak; seldom full and hard.

2. Chill and chilliness predominant, often as if cold water were poured over one, relieved by external warmth; evening and night chill alternating with heat; chill proceeding downward from the face or epigastrium (*Plexus solaris*), over the body, and chill beginning in the feet.

3. Heat often running over the body during the day; nocturnal attacks of flying heat, with great anxiety and restlessness.

4. Nocturnal debilitating sweat; one-sided sweat, mostly on the left side; sweat every other evening.

**Belladonna.**

1. Pulse quick, often full, hard, and tense, but sometimes small and soft; seldom slow, and then it is full. Throbbing of the carotids and temporal arteries.

2. Chill in the evening, especially on the extremities, most on the arms, with heat of the head; internal chill and external burning heat; chill alternating with heat. Evening, shaking chill, coldness of the limbs, with hot head; shuddering running down the back.

3. Constant dry, burning heat, with sweat only on the head; internal heat with anxiety and restlessness; heat of the forehead, with cold cheeks; internal or external heat, or both at the same time; heat of the head, with redness of face and delirium; heat generally predominating.

4. Sweat exclusively on covered parts; sweat with the heat, or immediately after it, mostly on the face; empyreumatic sweating, sweat which stains the linen; sweat during sleep, day and night, sweat wholly wanting, sweat rising from the feet to the head.

**Lycoperdon-bovista.**

1. Pulse excited, with ebullition of blood and palpitation of the heart.

2. Chill predominating, even by a warm stove, mornings, evenings, and even at night, generally with thirst; chill with the pains.

3. Daily, evening fever (about seven o'clock), consisting merely of chill with thirst; evening, shuddering proceeding from the back.

4. Every morning (from five to six o'clock) sweat most on the chest.

**Bryonia-alba.**

1. Pulse very full, hard, rapid, and tense, sometimes intermittent, with strong ebullition of blood.

2. Chill and coldness, predominant often with heat of head, red cheeks, and thirst; chill with external coldness of the body; chill and coldness, mostly evenings, and often only right-sided; chill more in a room than in the open air.

3. Dry, burning heat, mostly internal, as if the blood burned in the veins; during the heat all symptoms greatly increased.

4. Much sweat and sweating, very easily excited, even from slow walking in the open, cold air.

**Calcarea-carbonica.**

1. Pulse full and accelerated, and often trembling; much throbbing in the blood-vessels, and palpitation of the heart.

2. Chill, with shuddering, most evenings, but also forenoons; internal chilliness, mornings, after rising; chill alternating with heat.

3. Frequent attacks of flying heat, with anxious palpitation of the heart; heat, then chill, and cold hands; evening in bed external heat with internal chill; heat after eating.

4. Sweat from the slightest movement, even in cold open air; sweat in first sleep; morning sweat; sweats most on head and chest; sticky night-sweat only on legs.

**Camphora.**

1. Pulse small, weak, and slow, often imperceptible; diminished flow of blood to parts remote from the heart.

2. Chill, coldness, and sensitiveness to cold air; chill, with shuddering and shaking; universal icy coldness of the whole body, with deathly paleness of the face.

3. Heat, with swelling of the veins, increased by every movement.

4. Cold sweat, often sticky, and always debilitating.

**Cannabis-sativa.**

1. Pulse very weak, slow, often almost imperceptible.

2. Chill predominant, with thirst and shaking; external coldness of the whole body, except the face.

3. Heat only on the face; slight nocturnal burning heat.

4. Sweat wanting.

**Cantharis.**

1. Pulse very various, mostly hard, full and accelerated. sometimes intermitting; more seldom weak, slow, and almost imperceptible; pulsation through the trembling limbs.

2. Chill, with universal coldness, attacks mostly in the evenings, not relieved by external warmth; fever often consisting of chill, with subsequent thirst, without heat; chill running up the back.

3. Heat in the night, merely external, not sensible to patient; burning heat with anxiety and thirst.

4. Sweat from every movement; cold sweat, especially on hands and feet; sweat on genital organs; sweat has a urinous odor.

**Capsicum.**

1. Pulse very irregular and often intermitting.

2. Chill predominant, and almost always with great thirst; chill after every drinking; with shuddering; chill in cold air, especially in a current; evening chill; diminished natural heat of the body; sensation of cold sweat on the thighs.

3. Heat with co-existent sweat and thirst; internal heat with

